# The Importance of Considering Model Choices When Interpreting Results in Computational Neuroimaging

**DOI:** 10.1523/ENEURO.0196-19.2019

**Published:** 2019-12-13

**Authors:** Thomas C. Sprague, Geoffrey M. Boynton, John T. Serences

**Affiliations:** 1 Department of Psychological and Brain Sciences, University of California, Santa Barbara, Santa Barbara, CA 93106-9660; 2 Department of Psychology, University of Washington, Seattle, WA 98195-1525; 3 Department of Psychology, University of California San Diego, La Jolla, CA 92093-0109; 4 Neurosciences Graduate Program, University of California San Diego, La Jolla, CA 92093-0109; 5 Kavli Foundation for the Brain and Mind, University of California San Diego, La Jolla, CA 92093-0126

**Keywords:** computational neuroimaging, inverted encoding model, multivariate analysis, stimulus reconstruction

## Abstract

Model-based analyses open exciting opportunities for understanding neural information processing. In a commentary published in *eNeuro*, Gardner and Liu (2019) discuss the role of model specification in interpreting results derived from complex models of neural data. As a case study, they suggest that one such analysis, the inverted encoding model (IEM), should not be used to assay properties of “stimulus representations” because the ability to apply linear transformations at various stages of the analysis procedure renders results “arbitrary.” Here, we argue that the specification of all models is arbitrary to the extent that an experimenter makes choices based on current knowledge of the model system. However, the results derived from any given model, such as the reconstructed channel response profiles obtained from an IEM analysis, are uniquely defined and are arbitrary only in the sense that changes in the model can predictably change results. IEM-based channel response profiles should therefore not be considered arbitrary when the model is clearly specified and guided by our best understanding of neural population representations in the brain regions being analyzed. Intuitions derived from this case study are important to consider when interpreting results from all model-based analyses, which are similarly contingent upon the specification of the models used.

## Abstract (precis)

Model-based analyses open exciting opportunities for understanding neural information processing. In a commentary published in *eNeuro*, Gardner and Liu (2019) discuss the role of model specification in interpreting results derived from complex models of neural data.

## Significance Statement

Gardner and Liu (2019) point out that linear models can provide equally good fits to data across a class of linear transforms applied during analysis. They suggest this is particularly problematic for one analysis method, the inverted encoding model (IEM), that uses activation patterns to estimate responses in modeled information channels, as this renders results arbitrary. Instead, we argue results are not arbitrary when considered in the context of a well-motivated model. Of course, changing model properties can change results, but this applies to all model-based analyses, regardless of inversion. Changing properties of models to recover desired results without disclosure is always ill-advised. When used properly, especially to compare population-level response profiles across conditions, these approaches remain useful tools.

## Introduction

Scientists engaging in any research endeavor must necessarily make choices as they perform their research. One can select to measure scalp potentials with EEG or hemodynamic signals with fMRI; one can focus on neural responses in visual cortex or parietal cortex; and one can choose a particular set of modeling assumptions when analyzing data. In some cases, results robustly generalize across such choices. However, it is often the case that the choice made by the researcher matters when interpreting results. For example, scalp potentials index complementary types of attentional modulations to hemodynamic signals (Itthipuripat et al., 2019a), which can impact the conclusions of a study.

When using modern computational models to assay neural function, the modeling choices made by the researchers critically influence results of the modeling procedures. Therefore, it is impossible to interpret the results without knowledge of the details of the model used to generate those results. Moreover, altering the properties of the model should naturally change aspects of the results, sometimes in a predictable and straightforward way. This is true for all models, including the popular single-voxel population receptive field (vRF) modeling approach (Dumoulin and Wandell, 2008; Wandell and Winawer, 2015; Vo et al., 2017), fitting extremely high-dimensional voxel-wise encoding models to densely-sampled datasets (Kay et al., 2008; Naselaris et al., 2009; Nishimoto et al., 2011; Huth et al., 2012, 2016; Çukur et al., 2013; Lescroart and Gallant, 2019), the inverted encoding model (IEM) technique (Brouwer and Heeger, 2009, 2011, 2013; Scolari et al., 2012; Foster et al., 2016), and even fitting standard general linear models (GLMs) to task-based fMRI data (Friston et al., 1994).

In a recent commentary, Gardner and Liu (2019) focus on how choices about model specification are meaningful in the context of one of these techniques, the IEM. Typically, this technique involves experimenters estimating the parameters of a simplified model built of stimulus-selective feature channels (e.g., for orientation; color; motion direction; spatial position; polar angle), each tuned to specific feature values and tiling the full stimulus space (Freeman and Adelson, 1991). The properties of these channels are often inspired by our understanding of the visual system. There are populations of cells tuned to particular orientations; colors; motion directions; positions, and at least in early sensory areas, much is known about the characteristic shape of single-unit tuning functions, and how similarly tuned neurons are clustered along the cortical surface. As an example, one could build a model with eight channels tuned to different stimulus orientations, with each channel modeled with an orientation-selective circular Gaussian tuning function (Brouwer and Heeger, 2011; Ho et al., 2012; Scolari et al., 2012). Using these modeled channels, any stimulus generated from the modeled feature space can be described by the activation of the modeled channels. Then, based on the predicted responses of these modeled channels, linear regression is used to estimate how such a model accounts for changes in activation in each measured signal dimension, typically fMRI voxel or EEG electrode, across different stimulus conditions (fitting the “forward” model). The best-fit model can then be inverted to infer the activation of each modeled channel, that is, the reconstructed channel response profile, given the previously-estimated model and new measured activation patterns across many signal dimensions. The result, when channels are modeled as selective for a single stimulus value, is a channel response profile that typically exhibits a peaked response at the feature value(s) present in the stimulus. Importantly, the inversion step effectively summarizes the results by transforming modulations across all measured signal dimensions (e.g., all voxels or EEG electrodes) back into the model space.

While channel response profiles often look qualitatively similar to neural tuning functions for single units, a point brought up by Gardner and Liu (2019), it is critical to understand that reconstructed channel response profiles cannot be used to draw conclusive inferences about any specific attributes of single-unit response properties (e.g., single tuning width; for more on this, see Sprague et al., 2018a). Moreover, recovery of peaked channel response profiles does not demonstrate the accuracy (or inaccuracy) of the channel shapes in the encoding model used (Sprague et al., 2018a; Gardner and Liu, 2019). We note that Gardner and Liu (2019) use “channel response function” in their commentary, and others have used “channel tuning function,” to refer to results from the IEM technique; we elect to instead use “channel response profiles,” to further distance these results from single-neuron tuning functions.

In their commentary, Gardner and Liu (2019) argue that the channel response profiles resulting from the IEM technique are “arbitrary” because invertible linear transforms of the basis set will fit the data equally well. Hence, changing the shape of the modeled channels can predictably change the shape of the reconstructed channel response profiles. This ability to apply invertible linear transforms means that any reported channel response profile’s shape is one from an infinite family of shapes (spanned by all invertible linear transforms that could be applied to the analysis). In their words, “the channel response function is only determined up to an invertible linear transform. Thus, these channel response functions are arbitrary, one of an infinite family and therefore not a unique description of population representation.” (Gardner and Liu, 2019, their abstract). Thus, if a researcher used an unprincipled set of assumptions about the shape of the modeled channels (i.e. ignoring known properties of neural selectivity), then these assumptions can be recapitulated in the reconstructed channel response profiles. For example, Gardner and Liu (2019) showed that if orientation channels are presumed to be bimodal then the resulting reconstructed channel response profiles can also have a bimodal shape.

Below we argue that all models are arbitrary, even those informed by biology, but the results derived from the model are not arbitrary once the model has been specified. This is true for the IEM, but also other neural modeling approaches. Next, we show that even if poorly motivated models are used (or, equivalently, poorly motivated linear transforms are applied), differences between conditions assayed with the IEM technique can be preserved. Finally, we discuss important considerations when interpreting IEM-based analyses and what we see as the place for this modeling approach in the context of other useful analysis methods.

## IEM-Based Channel Response Profiles Are Uniquely Determined Given a Fixed Model

It is an unfortunate mischaracterization to imply that IEM-based results are “arbitrary” without specifying that they are uniquely determined and interpretable with knowledge of the encoding model basis used for analysis. Although one can generate many descriptions of a population representation, the result is not arbitrary if the channel response profile is interpreted in the context of the model used by the researchers. As a simple example, one invertible linear transform that could be applied to an encoding model basis and the resulting channel response profiles would shift the columns of the predicted channel response matrix by one. This would result in each channel being mislabeled, but all other features of the analysis would proceed intact. With knowledge of this mislabeling (that is, knowledge of the original basis and the invertible linear transform), it is possible to undo the transform and to achieve the intended understanding. Likewise, if the experimenter reports their basis (as all IEM reports do, so far as we know), and the reconstructed channel responses or derived measures are computed in the context of that basis, then there are no concerns as to the arbitrariness of the channel response profile’s shape. Thus, when principled model basis functions are chosen, it is appropriate to interpret the channel response profile as one possible, but not an arbitrary, depiction of the population representation, as uniquely derived given the principled model choices. That is, IEM results should not be interpreted as revealing *the* population representation; instead, they show one possible depiction of a population representation based on the particular model used.

## Results from All Models Depend on Properties of the Model

Importantly, the points Gardner and Liu (2019) raise about applying invertible linear transforms (that is, changing the coordinate system of a linear model) apply to nearly all model-based analyses, even those that only compute a forward encoding model to predict responses of measured neural signals based on stimulus properties, without any attempt at “inversion” back into a stimulus-referred space. We consider two trivial examples: spatial RFs measured via single-unit electrophysiology, and a GLM fit to a two-condition fMRI experiment.

When estimating the spatial RF of a neural measurement (either neuron or voxel), it is necessary to relate the observed neural response to changes in the stimulus. Under certain noise assumptions, one could even weight the stimulus aperture (in screen coordinates) by the observed neural signal. But even this procedure involves an implicit set of model assumptions, namely, that the basis for the stimulus model is in visual field coordinates (one number for each location in the visual field). Thus, the same logic of coordinate transforms applies here: one could apply any number of invertible linear transforms to the image basis and to the estimated RF profile, and the resulting model would account for the same amount of variance because it is a linear transform of the original model. For instance, a 2D Fourier transform could be used to losslessly transform between a spatial basis and a Fourier basis. Does this mean we should consider RF (or feature tuning) models as arbitrary? Of course not. The existence of a potential coordinate transform does not render the original model invalid, it just means that one must know the model to interpret the results.

A similar logic applies to a simple two-condition fMRI experiment using univariate statistical approaches (i.e., voxel-wise analysis with a GLM; Friston et al., 1994). Consider the case where a participant is sometimes pressing a button with their left hand and sometimes a button with their right hand (or looking at pictures of faces or houses, or any other experimental manipulation). The experimenter can build a GLM with predictors for BOLD activation associated with pressing a button with the left and right hand, appropriately convolved with a model hemodynamic response function. In turn, the experimenter could apply the invertible linear transform P = [0 1; 1 0] to the model basis (and thus, the resulting GLM regressors), which would result in flipped estimated β weights: the β weight originally corresponding to right now corresponds to left, and vice versa. However, because you know the original layout of the regressors, you could update your labels of the weights accordingly. While the ability to perform this coordinate transform in principle means the resulting β weights are arbitrarily defined, they remain uniquely and informatively defined given an understanding of the original model. This fact should not be used to label model-based estimates as arbitrary, but instead emphasizes the importance of understanding the model used to derive conclusions about a dataset.

Moreover, it is often the case that as understanding progresses, decisions about how to specify models change. This is evident among researchers developing and fitting voxel RF models: the original, classical demonstration that RF profiles can be fit for individual fMRI voxels implemented a straightforward circular Gaussian encoding model with an assumption of linear scaling (Dumoulin and Wandell, 2008). In subsequent studies, these models were extended to incorporate inhibitory surrounds (Zuiderbaan et al., 2012), compressive spatial nonlinearities (Kay et al., 2013; Mackey et al., 2017), and noncircular RF profiles (Silson et al., 2018), among many others. Does the introduction of a new model invalidate results using simpler models? Typically, it does not. While it is certainly the case that a more accurate model is always preferable, sometimes a simple model can still be useful (e.g., for defining boundaries of retinotopic ROIs). As always, the parameters reported are interpretable given understanding of the model used to derive those parameters.

## Differences between Conditions Are Preserved across Linear Transforms of the Basis

At a high level, the IEM technique is a form of model-based dimensionality reduction. This approach estimates a transform from idiosyncratic measurement space (e.g., activation in voxels in V1; α power at EEG scalp electrodes) into a principled, manipulable, model-based “information” space (activation across modeled information channels). Perhaps most importantly, many studies using IEMs seek to compare channel response profiles, or basis-weighted image reconstructions, across task conditions or timepoints in a trial. As described by Sprague et al. (2018a), these studies employ a fixed encoding model, such that activation patterns from different conditions are transformed into the same modeled information space, using a single common estimated encoding model (and often that encoding model is estimated using data from a completely different training task; Sprague et al., 2014, 2016, 2018b; Itthipuripat et al., 2019a,b). In this case, the criticisms raised by Liu et al. (2018) and Gardner and Liu (2019) do not apply: any arbitrary linear transforms would be applied equivalently to the results from each condition; and differences between conditions would be transformed from participant- and stimulus-specific measurement space into the same model-based information space. Invertible transforms would serve only to adjust the axes of the modeled information space, providing a different “view” of the same data. (Note that there may be cases where a transform renders differences between conditions invisible, but this would be exceedingly rare in cases where stimulus features span a feature space.)

**Figure 1.**
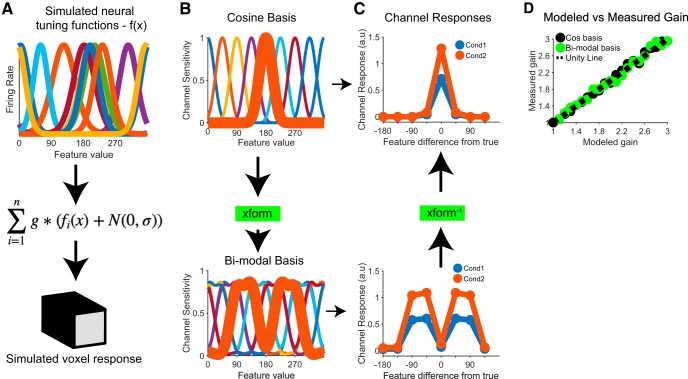
Differences between conditions can be preserved across invertible linear transforms. ***A***, We simulated voxel-level fMRI data where each voxel’s response was generated based on the sum of simulated responses across a population of simulated neurons with randomly centered tuning preferences and variable bandwidth (here, *n* = number of neurons, set to 100, although only 10 neural tuning functions are shown for clarity; see code on GitHub for full set of model parameters; https://github.com/tommysprague/iem_sim). Noise was added to the neural responses and then the gain factor (g) was applied to the data from each condition (condition 1: g = 1, condition 2: g = 1.8). For display purposes the noise (N) was set to 0 for panels ***A–C*** (following Gardner and Liu, 2019, their Fig. 3) and was set to 10 for panel ***D***. ***B***, We analyzed data using two different formats of channel basis functions, mirroring those used by Gardner and Liu (2019). Importantly, the two bases are related by an invertible linear transform (xform). ***C***, Reconstructed channel response profiles differ in similar ways: condition 2 has a higher amplitude than condition 1, regardless of the basis set used, and the bimodal channel response profiles are related by the inverse of the linear transform that was used to create the bimodal basis in the first place (xform^−1^). ***D***, Modeled gain compared to measured gain between conditions 2 and 1, computed using both the raised cosine basis set and the transformed bimodal version of the cosine basis set. Because there is not a straightforward way to quantify amplitude for the channel response profiles computed from the bimodal basis, we instead implemented a model-free quantification scheme in which we computed the ratio of the area under each channel response profile (i.e., ratio of area under the curve in condition 2 compared to condition 1).

To make more concrete the point that differences between conditions can be preserved across linear transforms of the basis, we simulated an fMRI dataset for an experiment that contained two conditions, with one condition evoking a multiplicatively-larger response at the underlying neural level than the other (e.g., an increase in contrast, as in Liu et al., 2018; code available at https://github.com/tommysprague/iem_sim). Briefly, the response of each of 100 simulated voxels was computed as the sum of the responses of simulated neurons within each voxel, with each simulated neuron having a circular Gaussian tuning function across the feature space (with pseudo-randomly determined tuning bandwidth and amplitude; Fig. 1*A*). The activity of the neurons within each voxel was computed in response to a set of 8 stimulus orientations across two experimental conditions, with multiplicative gain applied to the simulated neural responses in condition 2 compared to condition 1. One-half of the data, balanced across stimulus type and experimental condition, were designated as a training set and the other half of the data were designated as a testing set. Using data in the training set, we next fit the voxel-wise forward encoding model comprised of eight basis functions that span the feature space using either a standard set of raised cosine basis functions, tuned to specific feature values spanning the orientation space, or a set of raised cosine basis functions that were linearly transformed via an appropriately designed matrix into bimodal basis functions [termed the “xform” matrix (Fig. 1*B*); mirroring Gardner and Liu (2019)’s Fig. 2; the P matrix in their notation]. We then inverted both forward models, and used those IEMs to reconstruct channel response profiles from the same held-out test data.

Within each condition, channel response profiles recovered a scaled version of the basis function used to estimate the corresponding model (Fig. 1*C*; mirroring Gardner and Liu (2019)’s Fig. 3]. However, although the shape of the channel response profiles is constrained predictably by the choice of the basis functions, differences between conditions are preserved: condition 2 shows larger-amplitude channel response profiles regardless of the basis used. Importantly, because the transformation is linear and invertible, the bimodal channel response profiles from each condition can be losslessly converted back into unimodal channel response profiles via multiplication with the inverse of the original transformation matrix (Fig. 1*C*; and note that this holds across a variety of gain modulations and with noise added at the level of simulated neurons, Fig. 1*D*). Thus, one can apply arbitrary linear transforms to the basis set, and rather than rendering the data arbitrary, they remain interpretable given knowledge of the encoding model.

As shown in Figure 1, although the shape of the channel response profiles is different due to the application of an invertible linear transform, the difference between conditions is preserved. This follows from the fact that, because the end result of the IEM procedure is a linear mapping from signal space into channel space, some differences in measured signals can be detected even across arbitrary basis transforms.

Thus, if the goal is to determine whether the amplitude of the channel response profiles increased, then the application of an invertible linear transform should not impact the general conclusions. Of course, this is true so long as one can accurately quantify or parameterize the resulting shape of the channel response profiles, which may be difficult if a random or oddly-shaped basis is used. Similarly, the process of aligning or re-centering channel response profiles on the correct feature can become vaguely defined if poorly motivated basis functions are used: typically, a unimodal channel is centered at the feature value to which it is tuned; but a bimodal or other oddly-shaped channel cannot be easily related to a particular feature value, further rendering data presentation and interpretation tricky in such cases. But again, we emphasize that, when channel response profiles are interpreted within the context of the model used to compute them, there is no sense in which the reported result is arbitrary.

## Appropriately Selecting Neutral Training Sets

It is not always trivial to decide what type of data to use for estimating a fixed encoding model. Data SNR will likely vary across conditions, and fundamental properties of the encoding model itself may also vary (e.g., spatial RF properties; Vo et al., 2017). Because the IEM can be considered a form of model-based dimensionality reduction, it is quite important to estimate the most robust model possible. In general, using a neutral “mapping” dataset in which data SNR is optimized is, we believe, the most ideal approach, and can provide a stable “baseline” space in which to transform measured data from an experimental task. For example, when examining how working memory representations change across various task manipulations, we have used a variety of attended visual stimuli for model estimation (Sprague et al., 2016; Rademaker et al., 2019). This is analogous to common procedures used when identifying retinotopic maps on the cortical surface, where researchers typically employ high-contrast flickering checkerboard bars or wedges, and often ask participants to attend the visual stimulus in service of improving SNR (Bressler and Silver, 2010).

It is not always possible to acquire such mapping task datasets, either for experimental or scan time considerations. In this case, there are several possibilities. If multiple conditions are tested (e.g., conditions 1 and 2), one could (1) estimate an encoding model using data from both conditions 1 and 2 (equal numbers of trials), then use that encoding model to reconstruct held-out data from each condition (Sprague and Serences, 2013), or (2) estimate the encoding model with data from each condition in turn, then reconstruct data from both conditions 1 and 2 using each estimated encoding model. The key feature of such an analysis is that channel response profiles are compared based on a fixed encoding model. If different encoding models are used to reconstruct channel response profiles from different conditions, they may not be on equal footing (Liu et al., 2018), and it can be challenging to determine whether the best-fit encoding model changed, the structure of the neural representation changed, or both changed (Sprague et al., 2018a). In general, using a balanced dataset or the highest-SNR condition for model estimation should likely yield the most robust results, as these datasets will allow for accurate model estimation.

## Interpreting Channel Response Profiles

The points raised by Gardner and Liu (2019) offer an opportunity to clarify what can and what cannot be inferred based on results from IEM analyses. In general, the structure of the analysis constrains what can be concluded. We discuss two scenarios: reconstructing channel response profiles within-dataset and using a fixed encoding model.

### Computing channel response profiles within-dataset

Often, encoding models are estimated using a subset of runs or trials from an experiment, then the best-fit encoding model is used to reconstruct channel response profiles using the held-out runs or trials. In all other respects, the runs/trials used for model estimation and channel response profile reconstruction are identical. This is often called “leave-one-run-out” or “leave-one-trial-out,” or more generally, k-fold cross-validation. In such an analysis structure, the only interpretable result is the positive one: a reconstructed channel response profile peaked over the actual feature value (assuming a sensible model is used). If such a peak is observed after implementing appropriate statistical tests in which null models are used to compute channel response profiles, this offers evidence that the modeled feature value is represented within the measured neural signal space (i.e., ROI; EEG frequency band). That is, if one observes a smooth channel response profile in a leave-one-run-out analysis estimated using a smooth encoding model, this cannot and should not be taken as evidence for a smooth, graded representation of the modeled feature value within the analyzed data (Gardner and Liu, 2019).

However, there is one case where the shape of the channel response profile can be meaningful: if an orthogonal delta or “stick” basis is used, in which no smoothness is imposed on the encoding model, a graded channel response profile is consistent with a smooth representational structure, such that nearby stimulus feature values are represented more similarly in neural signals (Garcia et al., 2013; Saproo and Serences, 2014; Ester et al., 2015, 2016, Foster et al., 2016, 2017; Sutterer et al., 2019). This approximates a form of representational similarity analysis (RSA; Kriegeskorte et al., 2008; Kriegeskorte and Kievit, 2013). Note that this does not provide evidence for any particular single-unit tuning properties, but does support a smooth population-level representation as assayed with aggregate neural signals. Finally, in some circumstances, the delta basis can be considered a linear transform of any graded and overlapping basis set typically used, and so the model equivalence issues raised by Gardner and Liu (2019) indeed apply. But, this ability to transform between coordinate systems does not negate an observation of smooth channel response profiles estimated in the context of an orthogonal basis.

### Computing channel response profiles with a fixed encoding model

When it is feasible to acquire a unique model-estimation dataset that is only used for the purposes of fitting the encoding model, it is possible to make further conclusions based on the shape of reconstructed channel response profiles. In this analysis structure, the model estimation dataset is never used for reconstructing channel response profiles. Rather, separate “task” data in which one or more experimental conditions are manipulated is used for reconstruction. There is not necessarily any imposed structure on the resulting channel response profiles, and so their properties can be quantified and compared on a fair footing: the IEM fit using separate data offers a static perspective with which to view the measured activation patterns in the task data.

In the context of a fixed encoding model, the observation of peaked channel response profiles in the task dataset suggests that the information is represented in an analogous structure to its representation in the mapping dataset. For example, Rademaker et al. (2019) recently demonstrated that an encoding model estimated with an attentional mapping task (participants monitored an oriented grating for occasional contrast changes) could accurately recover the contents of visual working memory (participants remembered a briefly-presented oriented grating over an extended delay interval) based on activation patterns in occipital cortex.

### Changes in channel response profiles between conditions

Under a fixed encoding model, observing different channel response profiles between task conditions offers meaningful insight into the information content of population-level neural representations. Returning to the point that IEMs act as a form of model-based dimensionality reduction, the observation that channel response profiles change across task conditions suggests that the activation pattern is impacted by the experimental manipulation within the particular modeled information space. For example, manipulations of spatial attention are often found to impact the gain of channel response profiles (Garcia et al., 2013; Sprague and Serences, 2013; Itthipuripat et al., 2019a). Due to the linearity of the analysis, this is consistent with a neural mechanism whereby neural populations increase their gain with attention (Kim et al., 2007; Lee and Maunsell, 2010; Fig. 1). However, other mechanisms remain possible. As just one example, attention can impact the spatial RFs of neurons or voxels tuned to nearby locations (Womelsdorf et al., 2006), which can also contribute to an observed change in channel response profile amplitude (Sprague and Serences, 2013; Vo et al., 2017).

Changes in the baseline, width, or even center of channel response profiles can also be meaningfully compared across conditions under a fixed encoding model. For example, Ester et al. (2019) observed biased centers of channel response profiles following category learning, suggesting that human visual cortex represents similar feature values more distinctly when they belong to different learned abstract categories. Changes in the width of channel response profiles are more challenging to interpret, and less commonly observed, but these could be consistent with a uniform change in the tuning width of constituent neural signals (e.g., simulations in Liu et al., 2018). However, these changes are also consistent with other mechanisms, including asymmetric gain across feature channels (Scolari et al., 2012).

While it remains impossible to conclusively determine which mechanism(s) at the single neuron level support an observed change in channel response profiles, they can be compared when examined on equal footing using a fixed encoding model. Importantly, similar interpretational issues arise when considering changes in voxel RF parameters across task conditions (Klein et al., 2014; Kay et al., 2015; Vo et al., 2017), and changes in decoded neural uncertainty (Liu et al., 2018). Many types of changes at the unit level are consistent with observations at the level of single voxels and across large populations of voxels.

### What feature is represented?

Additionally, it is important to remember that identification of peaked channel response profiles (or, for that matter, successful decoding of stimulus value) does not unambiguously demonstrate that the modeled feature is represented by the brain signals measured. If a feature value could equivalently be written as a function of another feature, it remains possible that the analysis is sensitive to such confounding signals. For example, there have been several demonstrations that visual orientation (Freeman et al., 2011) and motion direction (Wang et al., 2014) are represented at a coarse scale in human visual cortex, such that the retinotopic position preference of a voxel determines its feature preference. In this case, it is possible that successful reconstruction or decoding of orientation or motion direction is a consequence of these confounded coarse retinotopic signals, rather than fine-grained feature-selective biases within individual voxels. In many cases this isomorphic feature mapping may not matter as experimenters simply want to characterize the representation of information encoded about a stimulus (in whatever format is accessible). However, it is always necessary to consider the relationship between different possible feature spaces when interpreting results from any model-based analysis of feature-selective neural response properties.

## Comparison of IEM and Bayesian Approaches to Stimulus Decoding

Gardner and Liu (2019) also make several other points. First, they highlight many positive aspects of the Bayesian decoding approach introduced by van Bergen et al. (2015). We agree that van Bergen and colleagues' (van Bergen et al., 2015; van Bergen and Jehee, 2018) use of a forward model combined with a Bayesian readout rule is an innovative and promising technique, and thoughtfully analyzing data in different ways, especially when employing complex models, is always a good idea. In particular, the Bayesian decoding approach can provide complementary information about the uncertainty with which the activation pattern represents a feature value using an independently-estimated noise model, which is especially useful when trying to link trial-by-trial readouts of neural uncertainty with behavioral measures (van Bergen et al., 2015; van Bergen and Jehee, 2019). That said, we note that the Bayesian approach, like all modeling endeavors, is sensitive to choices made by researchers. For example, analogous to how choices made about channel shapes can impact results from linear IEMs, choices made about the reduced noise model implemented in the Bayesian approach can substantially impact the results (van Bergen and Jehee, 2018). Moreover, invertible linear transforms of the basis set, if not accounted for during the estimation of noise covariance, can lead to large changes in decoding accuracy. As always, motivating analysis choices based on our understanding of neural systems, such as observations that noise covariance scales with tuning similarity (van Bergen and Jehee, 2018), will enable the most robust possible conclusions.

While the Bayesian approach offers many advantages, there are scenarios where directly comparing responses of modeled information channels is more informative. For example, Brouwer and Heeger (2011) compared responses at specific channels across contrast and stimulus conditions to evaluate the impact of cross-orientation suppression, and Ho et al. (2012) and Scolari et al. (2012) compared responses in channels tuned nearby the stimulus orientation across task (emphasize speed vs accuracy) and attention (target left vs target right) conditions. These types of analyses require examining the full response profile across all modeled channels, especially those that are not tuned to the presented stimulus value. This is not easily accomplished with typical decoding analyses, Bayesian or otherwise – that generate a point estimate of the most likely stimulus feature (with or without a corresponding estimate of uncertainty). Moreover, when trying to disentangle responses associated with simultaneously presented stimuli, specifying an appropriate model in the Bayesian framework is not always straightforward. It is necessary to explicitly define a concrete forward model for how multiple stimulus features interact, including, potentially, how they jointly impact the structure of correlated noise. This is certainly not a weakness of the Bayesian approach. It is always ideal to explicitly define model assumptions. However, sometimes it can be useful to visualize how activation patterns are altered within a simple and fixed linear model space, in which case the traditional IEM approach can be more appropriate.

## Instantaneous Decoding vs Extended Model Estimation

Here, we have primarily discussed the IEM technique and a Bayesian decoder based on a formally specified encoding model. There are several other techniques that have been applied to understand neural representations, including RSA (Kriegeskorte et al., 2008; Kriegeskorte and Kievit, 2013), voxel-wise encoding modeling (Kay et al., 2008; Naselaris et al., 2009, 2011; Nishimoto et al., 2011; Huth et al., 2012, 2016; Çukur et al., 2013; Lescroart and Gallant, 2019), and voxel RF modeling (Dumoulin and Wandell, 2008; Kay et al., 2013; Wandell and Winawer, 2015; Vo et al., 2017). These approaches can be broken down based on their goals: do we wish to build a comprehensive model of a stimulus’ representational space within a brain region, including the sensitivity profiles of constituent voxels? Or, do we instead prefer using a simplified model to make inferences about “instantaneous” brain states that can be compared across experimental conditions?

RSA, simplified vRF modeling, and voxel-wise modeling all require participants view stimuli spanning an entire stimulus space to estimate a single result (for RSA: a representational dissimilarity matrix; for voxel-wise modeling, the parameters to each voxel’s encoding model). For example, you cannot estimate a voxel’s RF profile with stimuli presented in a small number of positions (e.g., just one position on the left and one on the right side of the screen). Instead, you must present stimuli that span the entire stimulus space (e.g., the full extent of the screen, from top to bottom and side to side). Thus, to compare results from any of these methods between task conditions, one must exhaustively sample the stimulus space within each task condition independently. Several studies have taken this approach. Çukur et al. (2013) fit voxel-wise encoding models of semantic space to data acquired while participants viewed natural movies and reported the presence of faces or vehicles (in different scan runs). Accordingly, between scan runs, they could compare properties of the best-fit encoding models across voxels, and concluded that the structure of semantic space is warped between conditions. Several studies have manipulated the locus of spatial attention while participants view visual stimuli used to map vRFs. Klein et al. (2014) and Vo et al. (2017) required participants attend to fixed locations while presenting mapping stimuli, and Sprague and Serences (2013), Kay et al. (2015), Sheremata and Silver (2015), and van Es et al. (2018) all estimated voxel RF profiles during scanning runs when participants either attended to or ignored the mapping stimuli.

RSA and voxel-wise encoding models stand in contrast to the IEM, the Bayesian decoding method, and other decoding approaches. Using these methods, once an encoding model or decoder is estimated (on a held-out set of data), it can be applied to any new activation pattern, even on a time point-by-time point basis. One case in which this can be used is to compare channel response profiles across a large number of conditions, such as the location of spatial attention and stimulus contrast (Itthipuripat et al., 2019a). In this study, it was not necessary to evaluate channel response profiles at many different stimulus positions, so instantaneous estimates for a small number of stimulus positions offered an efficient means to determine the joint impacts of attention and contrast on population-level stimulus representations. Put another way: there is no way to estimate a voxel-wise encoding model, vRF profile, or representational dissimilarity matrix on a single trial. If single-trial analyses are critical for a given research question, applying an independently-estimated encoding model or decoder is necessary. If, instead, a careful assay of the representational geometry and/or encoding properties of a neural signal are important, analysis of best-fit encoding models to extended datasets should be used.

Finally, the two approaches might be integrated: if trying to understand how a poorly-understood feature space is represented on individual trials, it may be best to start by inferring representational geometry with modern versions of techniques like RSA (Kriegeskorte et al., 2008; Kriegeskorte and Kievit, 2013; Walther et al., 2016; Cai et al., 2019), then using features of the inferred geometry in combination with model-based analysis methods like IEM or a Bayesian decoder applied to a separate dataset to recover trial-by-trial representations of feature information.

## Units of Channel Response Profiles

Gardner and Liu (2019) also point out that the units of model-based reconstructions are arbitrary. This is a point that was noted in one of the original papers to use an IEM (Brouwer and Heeger, 2011). We agree that reconstructed channel response levels are in arbitrary units, and we recommend researchers report them as such going forward. This, combined with the use of unit-normalized modeled channels (i.e., those used to predict channel responses when fitting the forward model), will render channel response estimates more comparable across studies. That said, it is essential to note that these units have no impact on the inferences that can be drawn when comparing channel response functions between conditions under a fixed encoding model. Thus, Gardner and Liu (2019)’s concerns about the arbitrary nature of this scale are not particularly germane to the interpretation of such results: one could scale all units by 42 without impacting the difference between conditions. Thus, so long as all model-based reconstructions that are compared head-to-head are on the same initial footing, then the comparisons are valid regardless of the conventions used to label the units of this analysis. However, when different models are trained for different conditions, it is less certain how to interpret differences in reconstructed channel response profiles across conditions: did the best-fit model, fit individually to each condition, change? Did the data used to reconstruct channel response profiles change? Did both change? By holding at least one aspect constant (the model, estimated with a neutral task or in a balanced fashion across conditions), it is possible to better ascertain how certain properties of neural response patterns change based on stimulus or task conditions as the units can be compared on equal footing (Sprague et al., 2018a).

## IEMs, and Other Analyses Applied to Voxel-Based Measurements, Cannot Be Used to Infer Properties of Single-Unit Tuning

Finally, Gardner and Liu (2019) and Liu et al. (2018) imply that one of the goals of the IEM is to make inferences about single neuron response properties. Making inferences about the response properties of single-neurons is not possible using the IEM or any related model that operates at the scale of aggregate neural signals such as voxels, as different types of single-unit modulations can give rise to identical modulations at the level of a voxel (Sprague et al., 2018a). As described above (see Interpreting Channel Response Profiles), divergent types of changes in single-neuron response properties can lead to identical signals at the aggregate scale. Thus, making such inferences is not the goal of the IEM or related measures, including the Bayesian decoding approach of van Bergen et al. (2015). Instead, a fundamentally different approach that likely requires adopting a different measurement/analysis paradigm, such as parallel characterization of response properties measured across different scales (e.g., fMRI BOLD signal and single-unit electrophysiology; Keliris et al., 2019) would be needed to overcome the ill-posed many-single-neurons-to-voxel mapping problem.

## Defining Terms

In the spirit of Gardner and Liu (2019)’s and Liu et al. (2018)’s efforts to delineate the appropriate uses of IEMs, we want to more precisely define several terms related to the IEM technique to help clarify future reports. The IEM technique involves estimating an *encoding model* that best accounts for observed voxel activation responses given stimuli that are transformed into a modeled “channel space” (and under the assumption of linearity such that the response of a given voxel is a linear combination of each of several modeled channels). Once an encoding model is estimated separately for each voxel, that encoding model can be *inverted* and used to *reconstruct* channel response profiles given new measured activation patterns across those same voxels. Those activation patterns are often measured in response to some kind of stimulus (either visual, or something attended, or held in working memory), and the resulting reconstructed channel response profiles typically contain representations of the stimulus/stimuli. To be clear, the result is not strictly a “stimulus reconstruction,” but a model-based reconstructed channel response profile. As an example, reconstructed channel response profiles for stimulus orientation are not literally an oriented grating. Instead, they describe the activation of modeled channels in response to a given stimulus, and this description is in a stimulus-referred space. Reconstructed channel response profiles can be used for several purposes, including *decoding* (recovering the most likely feature value(s) represented, and/or, with the use of an appropriate noise model, their uncertainty) and *quantification* (characterizing the shape of the channel response profile, including “width,” “amplitude,” etc., which should never be confused with the width or amplitude of single-neuron responses). Of course, all quantification of channel response profiles must be considered in concert with the encoding model used, but if a fixed encoding model is used for reconstructing channel response profiles across several experimental conditions, their properties can be compared in the context of the model.

## Conclusions

In this reply to Gardner and Liu (2019), we hope to have clarified some mischaracterizations of how the IEM approach is conducted (see also: Sprague et al., 2018a). To be clear, we are not arguing that the IEM or related approaches are not without serious limitations, the model specification is key, as is understanding what inferences can and cannot be supported by the results (Sprague et al., 2015, 2018a). As Gardner and Liu (2019) point out, these limitations are especially important to recognize when modeling signals in feature spaces that are not well understood, such as those for complex shapes or for higher-order cognitive or social functions. In these situations, an IEM may still be able to quantify differences between conditions and could thus be used to make inferences about changes in the information content of population-level response patterns. However, in this context, drawing unambiguous links between the shape of IEM-derived channel response profiles and the properties of population-level neural representations is not appropriate because changes in the model can result in changes in channel response profiles. Instead, we agree with the suggestions of Gardner and Liu (2019) that careful comparison of forward models that are not related by an invertible linear transform is better suited for this purpose (Brouwer and Heeger, 2009; Nishimoto et al., 2011; Lescroart and Gallant, 2019). That said, IEM-based channel response profiles are not arbitrary when the model choice is based on principled assumptions about neural population representations and, more importantly, channel response profiles are uniquely determined given knowledge of the modeled basis, whatever that basis may be. Finally, similar interpretational issues arise across many types of model-based analyses of neural signals, including voxel RF modeling, voxel-wise encoding models, and RSA. For all these approaches, modeling choices have substantial impacts on results, and so all results must always be interpreted in the context of the model(s) used for analysis.

We believe the IEM method is most useful when comparing reconstructed channel response profiles across manipulations of stimulus properties (e.g., contrast) or task conditions (e.g., attention), or combinations thereof (Sprague et al., 2018b) using a fixed encoding model across relevant comparisons (Sprague et al., 2018a). When used this way, the criticisms raised by Gardner and Liu (2019) have no substantial bearing on the efficacy of the IEM technique for comparing the impact of experimental manipulations on information represented within aggregate measurements of neural activity patterns. In other words, in the same way the answer (42) is only meaningful in the context of the question (Adams, 1979, 1981), results derived from a model are only meaningful in the context of the model used.

## Synthesis

Reviewing Editor: Li Li, New York University Shanghai Decisions are customarily a result of the Reviewing Editor and the peer reviewers coming together and discussing their recommendations until a consensus is reached. When revisions are invited, a fact-based synthesis statement explaining their decision and outlining what is needed to prepare a revision will be listed below. The following reviewer(s) agreed to reveal their identity: Won Mok Shim, Nihong Chen.

I invited four reviewers for this ms and the reviews from these four reviewers are very consistent. Three recommended minor revision and one recommended accept as it. I synthesize their reviews below. We will consider the publication of this ms after the concerns are addressed.

Reviewer 1:

This manuscript aims to settle an ongoing debate in the literature regarding the 'Inverse Encoding Model' (IEM) framework -an approach that's taken the field by storm in recent years. Mild controversy has emerged around this analysis approach, with some folks calling into question the conclusions that can be drawn from results using IEM's. There's been a healthy back and forth between certain camps. Some have warned that those drawing conclusions from IEM analyses should proceed with caution, due to the “arbitrary” nature of model selection/outcomes. This has served as an important gentle warning regarding potential (mis)interpretation of IEM results. In response, however, the authors of this current piece put forth a number compelling arguments in defense of the IEM approach, and I'm inclined to agree with the authors; there's no reason to throw the baby out with the bathwater. Specifically, the authors contend that a number of the critiques leveled against the IEM approach are the same critiques that could be leveled against the bulk of modeling endeavors. Namely, the nuts and bolts of models are only arbitrary to the degree to which they are or are not sensibly informed. Moreover, they argue that this method is primarily useful for examining *changes* in the reconstructed responses, and that these changes are preserved across linear transforms of the basis functions. Finally, they again reiterate that the reconstructed representation is an estimate of the population response, and in no way is it representative of single-unit responses. This piece is generally well written, and I think will help bring the key aspects of this debate into better focus. There are, however, a few key points that I think the authors could expand on to further clarify things.

1. While the authors agree that the selection of any channel shape would yield a recoverable channel response with that seemingly “arbitrary” shape, they rightfully point out that the choice of unimodal basis functions that resemble a bank of orientation-tuned filters to represent V1 would hardly be considered arbitrary. I agree. However, the IEM approach has been employed well outside of primary visual cortex, with applications often extending along large swaths of the dorsal stream, and even frontal portions of cortex. Yet, the precise selectivity profiles within many of these regions are, at best, in their nascent stages of understanding. It's doubtful that voxels within frontal cortices possess a clean bank of orientation selective filters (much to the chagrin of all vision scientists). That said, previous work that has explored these territories with the IEM approach have usually carried out the appropriate statistics, such as permutation tests, to demonstrate that the ability to recover channel responses is not spurious. So what, then, are readers supposed to interpret from those results? The authors should elaborate on how one should interpret results in areas where we do not have clearly informed basis functions. Are we supposed to simply interpret this as evidence only for, say, “orientation information” within these regions? Acknowledgement of this discrepancy, and a prescriptive roadmap for how to proceed with interpretations within these areas seems necessary.

2. One of the central points put forth by Gardner & Liu is that the selection of basis function determines the qualitative shape of the resulting inverted channel tuning function. Like I've mentioned above, the authors of the current piece have done a good job motivating their particular selection of basis function shape, especially within early visual cortices. One thing that I'm left wondering about, though, is what one is supposed to interpret from the *shape* of the resulting channel response profile. In particular, what would give rise to, for instance, a change in the width (or fidelity) of this function, that would be dissociable from a change in the amplitude? If, for instance, with the IEM approach, line observed that the width of a channel tuning function changed with some cognitive manipulation, does that arise from modulatory effects that can be interpreted as different in underlying source from a change in overall gain? Are there simulations that could be provided to shed light on the dissociation between width changes and amplitude changes? Or does it all boil down to SNR, with the source of modulation unclear, rendering the shape of the function uninterpretable in of itself? And if that's the case, why would one opt for this approach instead of some variant of classification or bayesian approaches to stimulus decoding? In generally, it would be useful for the authors to expand more on how one is supposed to interpret the output of an IEM analysis. Do we make anything out of changes in the shape?

3. In the last sentence, the authors forgot a citation (Adams, 1978)

Reviewer 2:

This paper has addressed important theoretical and interpretational issues on the Inverted Encoding Model (IEM) raised by Gardner and Liu (2019), and I believe this paper will contribute to clearing up some confusions about the IEM and stimulate productive debates.

I have some comments about the points that can be strengthened.

1. The authors wrote “whether an analysis is recovering the hypothetical responses of an arbitrary model rather than assessing the selectivity of population representations is not an issue unique to the inverted encoding model and human neuroscience, but a general problem that must be confronted as more complex analyses intervene between measurement of population activity and presentation of data.” I strongly agree that all modeling studies are built upon a set of assumptions, which is inevitable given the measurements and the prior knowledge, and the IEM is not unique in that regard. In this context, I think the authors could strengthen their argument if they provided an example(s) from other model-based approaches, such as the Bayesian decoding approach (Van Bergen et al., 2015). For instance, the Bayesian decoding approach also relies on assumptions about the noise covariance structures. Just like the IEM, the authors of the Bayesian decoding approach used their best prior knowledge about the noise structure, and the results would be inevitably dependent on how the noise structure is assumed in the model. The authors' argument would be more convincing if they showed a concrete example other than the case of an IEM.

2. The authors listed the strengths and weaknesses of the Bayesian approaches in comparison with the IEM. One of the points they mentioned is that “when trying to disentangle responses associated with simultaneously presented stimuli, specifying an appropriate model in the Bayesian framework is not necessarily straightforward.” I wish that the authors had clarified why this is particularly problematic for the Bayesian approach and why the IEM would suffer less from the same problem.

3. The authors mentioned the necessity of using a “fixed encoding model” when comparing different experimental conditions. However, it is not always clear how to build an encoding model that is neutral to all experimental conditions. Even if independent datasets where a different task was performed are acquired for model building, the resulting model could be biased toward one or another condition depending on what cognitive processes are required in the model-building dataset and how similar these processes are to those involved in the main experimental conditions. If we combine the equal amount of data from each experimental condition to build the model, it may still be a problem as the data from the condition with higher SNR are weighted more compared to other condition(s). It would be informative if the authors addressed this issue and suggested a way to mitigate the problem.

4. There are many recent papers on a range of topics using the IEM (see below for a few examples) other than those mentioned in the manuscript. The authors may want to check out these references and include them if relevant.

Chen N, Bi T, Zhou T, Li S, Liu Z, Fang F (2015) Sharpened cortical tuning and enhanced cortico-cortical communication contribute to the long-term neural mechanisms of visual motion perceptual learning. Neuroimage 115:17-29.

Chong E, Familiar AM, Shim WM (2016) Reconstructing representations of dynamic visual objects in early visual cortex. Proc Natl Acad Sci USA 113:1453-1458.

Lorenc ES, Sreenivasan KK, Nee DE, Vandenbroucke ARE, D'Esposito M (2018) Flexible coding of visual working memory representations during distraction. J Neurosci 38:5267-5276.

Minor point:

Page 5: Fig. 2 and Figure 2C should be Fig. 1 and Figure 1C, respectively.

Reviewer 3:

This is an enjoyable response to a valid set of criticisms by Gardner and Liu. Together, the two commentaries (original+response) serve to claify an important set of issues relating to the use of IEMs. I think, frankly, that the two groups are more or less in agreement at this point but this was a useful exchange of views. I wonder if it might be possible for both parties to collaborate on a review paper outlining 'best practises' for the use of IEMs - identifying common pitfalls and illustrating how they can be used in a principled manner for hypothesis testing?

Reviewer 4:

As a response to the commentary by Gardner & Liu (2019), this paper claimed that the inverted encoding model should not be considered arbitrary when the model is properly specified. Even if poorly motivated models are used, differences between conditions are generally preserved. The inverted encoding model remains a useful tool for comparing population-level response profiles across conditions.

The clarifications on what can be and what cannot be drawn from the IEM model are important for future research. I hope my questions and comments can help to further enhance its clarity.

1.

The authors clarified that IEM technique is a form of model-based dimensionality reduction, and making inferences about the response properties of single-neurons is not possible using the IEM. How about the inference at a population level? To what extent can the reconstructed response profiles reflect the underlying aggregate neural response change (i.e. baseline change, amplitude change, and bandwidth change) across different conditions?

2.

In the section of model comparison, the differences between IEM and Bayesian approaches were not stated clearly. E.g. “These types of analyses require estimating a full response profile across modeled channels, which is not easily accomplished with decoding analyses that generate a point estimate of the most likely stimulus feature....”. What is “full response profile”? Why is it not easily accomplished with decoding analyses? Also, the cases discussed were about the feasibility of IEM over the Bayesian approach, but not “scenarios when directly comparing responses of modeled information channels can be informative”.

3.

The authors listed a number of modeling approaches, e.g. representational similarity analysis, voxel-wise encoding modeling using naturalistic stimuli, simplified voxel RF modeling using specific stimulus sets. It would be valuable to discuss the applicable scenario for each modeling approach, and also the advantages and disadvantages of IEM over these modeling approaches.

## Author Response

Synthesis Statement for Author (Required): I invited four reviewers for this ms and the reviews from these four reviewers are very consistent. Three recommended minor revision and one recommended accept as it. I synthesize their reviews below. We will consider the publication of this ms after the concerns are addressed. We appreciate the thorough evaluation by the 4 reviewers, and address each of their comments in detail below. The reviewers' comments, in full, appear in bold, and our replies appear in plain text. New manuscript sections are quoted in italics. Reviewer 1: This manuscript aims to settle an ongoing debate in the literature regarding the 'Inverse Encoding Model' (IEM) framework -an approach that's taken the field by storm in recent years. Mild controversy has emerged around this analysis approach, with some folks calling into question the conclusions that can be drawn from results using IEM's. There's been a healthy back and forth between certain camps. Some have warned that those drawing conclusions from IEM analyses should proceed with caution, due to the “arbitrary” nature of model selection/outcomes. This has served as an important gentle warning regarding potential (mis)interpretation of IEM results. In response, however, the authors of this current piece put forth a number compelling arguments in defense of the IEM approach, and I'm inclined to agree with the authors; there's no reason to throw the baby out with the bathwater. Specifically, the authors contend that a number of the critiques leveled against the IEM approach are the same critiques that could be leveled against the bulk of modeling endeavors. Namely, the nuts and bolts of models are only arbitrary to the degree to which they are or are not sensibly informed. Moreover, they argue that this method is primarily useful for examining *changes* in the reconstructed responses, and that these changes are preserved across linear transforms of the basis functions. Finally, they again reiterate that the reconstructed representation is an estimate of the population response, and in no way is it representative of single-unit responses. This piece is generally well written, and I think will help bring the key aspects of this debate into better focus. There are, however, a few key points that I think the authors could expand on to further clarify things. We appreciate the reviewer's thoughtful comments, and are glad they believe this work helps focus the ongoing debate. We hope the updated manuscript helps clarify the issues pointed out by the reviewer. 1. While the authors agree that the selection of any channel shape would yield a recoverable channel response with that seemingly “arbitrary” shape, they rightfully point out that the choice of unimodal basis functions that resemble a bank of orientation-tuned filters to represent V1 would hardly be considered arbitrary. I agree. However, the IEM approach has been employed well outside of primary visual cortex, with applications often extending along large swaths of the dorsal stream, and even frontal portions of cortex. Yet, the precise selectivity profiles within many of these regions are, at best, in their nascent stages of understanding. It's doubtful that voxels within frontal cortices possess a clean bank of orientation selective filters (much to the chagrin of all vision scientists). That said, previous work that has explored these territories with the IEM approach have usually carried out the appropriate statistics, such as permutation tests, to demonstrate that the ability to recover channel responses is not spurious. So what, then, are readers supposed to interpret from those results? The authors should elaborate on how one should interpret results in areas where we do not have clearly informed basis functions. Are we supposed to simply interpret this as evidence only for, say, “orientation information” within these regions? Acknowledgement of this discrepancy, and a prescriptive roadmap for how to proceed with interpretations within these areas seems necessary. The reviewer brings up an excellent and important point. The IEM analysis framework is best suited for cases where we have some amount of understanding of the underlying representational structure, typically

based on studies probing single-unit neural tuning. However, it has additionally been applied in several scenarios outside this prescription (e.g., Ester et al, 2015; 2016), to evaluate 'sensory-like' codes for stimulus features like orientation present in non-sensory brain regions, such as parietal and frontal cortex. There are two types of scenarios for using IEMs that enable different sorts of interpretations. First, Scenario 1: if a study estimates an encoding model using the same dataset as used for reconstructing channel response profiles (employing an appropriate cross-validation scheme), the only possible interpretation afforded by observation of a peaked channel response profile is that there is activation across the measured voxel population that changes as a function of stimulus feature value(s). Per the arguments clearly outlined by Gardner & Liu (2019), one cannot use the shape of the resulting channel response profile to make any clear conclusions about the encoding properties of neurons or even populations in the region. Thus, peaked channel response profiles are more or less analogous in this scenario to an above-chance classification accuracy using a linear decoder like a support vector machine (SVM). However, there is one important wrinkle here: many studies evaluate encoding models that do not impose any correlated structure on the modeled information channels - instead, they use orthogonal delta or 'stick' functions (e.g., Scolari et al, 2012; Saproo & Serences, 2014; Ester et al, 2015; 2016; Foster et al, 2016). If the resulting channel response profile is 'smooth', then this provides some evidence that there is greater similarity in neural response patterns for more-similar feature values than for more-dissimilar feature values. As such, this approximates a “representational (dis)similarity analysis” (Klevit & Kreigeskorte, 2013). Such a smooth structure, when interpreted in the context of a stick-function model, can be considered meaningful, and can provide support for a smooth representational geometry in the space of the measured voxel activation pattern. Accordingly, observation of such structure can motivate subsequent analyses using 'smooth' basis functions (but note that, in many cases, smooth basis functions are related to delta functions by an invertible linear transform). Overall, for developing understanding of representational geometry, we recommend applying modern forms of representational similarity analyses (RSA; Cai et al, 2019; Walther et al, 2016). With an understanding of this geometry in hand, encoding models might be applied to understand instantaneous stimulus representations as parameterized in a model-based information space. The second common type of IEM analysis - Scenario 2 - involves estimating a model using a 'neutral' set of data, either one aggregated over several conditions (e.g., Sprague & Serences, 2013), or one reserved for such a model estimation purpose (e.g., Sprague et al, 2014; 2016; 2018; Rademaker et al, 2019; Itthipuripat et al, 2019), and applying this encoding model to reconstruct channel response profiles from activation patterns measured during several task conditions. Throughout this manuscript and a previous commentary (Sprague et al, 2018) we have referred to this as using a 'fixed encoding model'. In this case, especially when an entirely separate 'mapping' dataset is used for model estimation, there is no foregone conclusion as to what the reconstructed channel response profiles might look like for the task conditions in question. For example, this is particularly evident in studies in which we have reconstructed channel response profiles using data measured while participants viewed/remembered multiple visual stimuli (Sprague et al, 2014; 2016; 2018; Itthipuripat et al, 2019), or simultaneously viewed and remembered different stimuli (Rademaker et al, 2019). The observation of multiple peaks in reconstructed channel response profiles is not in any way built in to the analysis, and their observed structure can be used (with knowledge of the modeled information channels) to better understand how large-scale neural populations simultaneously represent multiple stimuli or stimulus features simultaneously. Finally, it is important to acknowledge that successful model-based reconstruction (or, for that matter, decoding) of a stimulus feature value does not necessarily prove that the modeled/decoded stimulus feature itself is represented by the observed neural activation. Instead, if another feature value unambiguously covaries with the modeled feature value, it remains possible that the analysis is in fact sensitive to the confounding feature rather than the modeled feature. This issue has been raised to address successful decoding of orientation (Freeman et al, 2011; 2013; Roth, Heeger & Merriam, 2018) and motion direction (Wang et al, 2014) based on visual cortex activation patterns: in some cases, the analyses may be sensitive to coarse-scale biases in feature selectivity across retinotopic maps, such that the decoder is actually picking up on spatial signals, rather than fine-scale feature-selectivity (though the contribution of fine-scale

signals is also supported, e.g., Alink et al, 2013; 2017; Pratte et al, 2016). In many cases, such signals can still be considered useful for assaying the impact of cognitive manipulations on the information content of large-scale activation patterns. But, the possibility of isomorphism between feature spaces must always be considered when interpreting results from any model-based analysis of feature-selective neural response properties. We have added an extensive new section to the manuscript, subtitled “Interpreting channel response profiles,” discussing these important interpretational issues (pgs 9-12; not copied here for brevity).

2. One of the central points put forth by Gardner & Liu is that the selection of basis function determines the qualitative shape of the resulting inverted channel tuning function. Like I've mentioned above, the authors of the current piece have done a good job motivating their particular selection of basis function shape, especially within early visual cortices. One thing that I'm left wondering about, though, is what one is supposed to interpret from the *shape* of the resulting channel response profile. In particular, what would give rise to, for instance, a change in the width (or fidelity) of this function, that would be dissociable from a change in the amplitude? If, for instance, with the IEM approach, line observed that the width of a channel tuning function changed with some cognitive manipulation, does that arise from modulatory effects that can be interpreted as different in underlying source from a change in overall gain? Are there simulations that could be provided to shed light on the dissociation between width changes and amplitude changes? Or does it all boil down to SNR, with the source of modulation unclear, rendering the shape of the function uninterpretable in of itself? And if that's the case, why would one opt for this approach instead of some variant of classification or bayesian approaches to stimulus decoding? In generally, it would be useful for the authors to expand more on how one is supposed to interpret the output of an IEM analysis. Do we make anything out of changes in the shape? These are all excellent questions. To return to the two scenarios described in our response to Comment 1: Scenario 1: leave-some-data-out cross-validation within-dataset - in this case, the shape of the channel response profile is almost entirely governed by the model choices made by the experimenter, with the same caveat that 'smooth' channel response profiles for models with non-overlapping 'stick function' channels are supportive of a smooth representational geometry. Otherwise, it is very difficult to make concrete conclusions based on the shape of the channel response profile, exemplified very clearly by the simulations conducted by Liu, Cable & Gardner (2018). Indeed, even comparing reconstructed channel response profiles (computed using within-dataset cross-validation) across regions of interest or task conditions is challenging, since differences in the number of voxels and SNR can impact the shape of the channel response profile in complicated ways (both at model estimation and at reconstruction; this may contribute to the empirical results shown in Liu et al, 2018). Scenario 2: estimate a fixed model that's used for reconstruction of data across conditions, which are subsequently compared to one another: in this case, changes in the channel response profile are certainly meaningful. For this scenario, IEM-based analyses are a form of model-based dimensionality reduction. When we change a task parameter, like stimulus contrast or attention, we measure a new activation pattern in signal (voxel) space. The IEM analysis then projects this new activation pattern into our model-based information (channel) space. If the change in activation pattern results in a different channel response profile, this suggests that the population response changed within our model-based information space. It is important to note that this is not necessarily the case: it is certainly possible (but usually unlikely) for the measured activation pattern to change along a trajectory that lies in the nullspace of the inverted encoding model, such that the reconstructed channel response profile remains stable across conditions.

Within Scenario 2, there are several possible changes in channel response profiles we might observe. A typical observation when using IEMs for spatial locations (e.g., Sprague & Serences, 2013; Itthipuripat, Sprague & Serences, 2019; Itthipuripat et al, 2019) is that the amplitude of a stimulus representation within the reconstructed channel response profile scales with attention. Per the linearity of the analyses, this is consistent with applying a scalar multiple to the activation profile across an ROI, which would propagate through the IEM to the reconstructed channel response profile. While this is not necessarily what is occurring at the neural or voxel level, an observation of amplitude changes is consistent with such a mechanism. But, amplitude changes are also consistent with a complex plethora of changes at the singleunit level, including, but not limited to, changes in RF position, changes in RF size (Vo, Sprague, Serences, 2017) additive scaling of response in some neurons but not others, divergent changes in response baseline across neurons within a voxel, etc. Another plausible change in channel response profiles is a widening or narrowing of the stimulus representation. Such a change is more complicated to interpret, and is subject to the same challenges for directly inferring the supporting neural mechanism. However, if channels tuned towards a presented stimulus respond more strongly and those tuned away respond more weakly, this remains meaningful. Another possibility, though not commonly reported, is for a stimulus representation to shift within the channel response profile. We've recently seen evidence of this following category learning (Ester et al, pp2019), and the results of Scolari et al, 2012 could be interpreted this way. If this result is observed under a fixed encoding model, then it is possible to make inferences about the represented feature value by the population across conditions. Overall, when a fixed encoding model is used for reconstructing channel response profiles, any observed change can and should be considered meaningful. Finally, despite these interpretational issues, it's necessary to point out that similar issues regarding the neural mechanisms supporting changes across conditions exist within other model-based analyses, including comparing voxel receptive field models across task conditions and applying the Bayesian framework to decode stimulus likelihood functions. Within the Bayesian framework, if you see a change in neural uncertainty across conditions, this could be due to a change in the 'amplitude' of the population response, a change in the noise properties, or a change in the underlying tuning width (or all of the above!). As described in a previous commentary (Sprague et al, 2018), the analysis method itself is irrelevant - it is never possible to make unambiguous inferences about constituent neural contributors to an aggregate neural signal. The Bayesian extension to the linear IEM does not resolve this ambiguity, but may allow for more useful quantification of neural activation patterns in some situations. Transforming channel response profiles into likelihoods based on an estimated noise model may confer greater explanatory utility, as neural uncertainty is concretely defined in stimulus units (thus far, as the standard deviation of the likelihood function over stimulus values). But, for other questions, such as studies interested in understanding how different neural populations may change their responses across stimulus and task manipulations, comparing channel response profiles may be a more useful approach as one has access to the entire response profile as opposed to a decoded estimate of the represented feature value and its uncertainty. We have added text to the manuscript to address these issues in the new section “Changes in channel response profiles between conditions (pg 11):

”Under a fixed encoding model, observing different channel response profiles between task conditions offers meaningful insight into the information content of population-level neural representations. Returning to the point that IEMs act as a form of model-based dimensionality reduction, the observation that channel response profiles change across task conditions suggests that the activation pattern is impacted by the experimental manipulation within the particular modeled information space. For example, manipulations of spatial attention are often found to impact the gain of channel response profiles (e.g., Garcia et al., 2013; Sprague and Serences, 2013; Itthipuripat et al., 2019a). Due to the linearity of the analysis, this is consistent with a neural mechanism whereby neural populations increase their gain with attention (Kim et al., 2007; Lee and Maunsell, 2010; Fig. 1). However, other mechanisms remain possible. As just one example, attention can impact the spatial RFs of neurons or voxels tuned to nearby locations (Womelsdorf et al., 2006), which can also contribute to an observed change in channel response profile amplitude (e.g., Sprague and Serences, 2013; Vo et al., 2017).

Changes in the baseline, width, or even center of channel response profiles can also be meaningfully compared across conditions under a fixed encoding model. For example, Ester et al (pp2019) observed biased centers of channel response profiles following category learning, suggesting that human visual cortex represents similar feature values more distinctly when they belong to different learned abstract categories. Changes in the width of channel response profiles are more challenging to interpret, and less commonly observed, but these could be consistent with a uniform change in the tuning width of constituent neural signals (e.g., simulations in Liu et al., 2018). However, these changes are also consistent with other mechanisms, including asymmetric gain across feature channels (e.g., Scolari et al., 2012).

While it remains impossible to conclusively determine which mechanism(s) at the single neuron level support an observed change in channel response profiles, they can be compared when examined on equal footing using a fixed encoding model. Importantly, similar interpretational issues arise when considering changes in voxel RF parameters across task conditions (e.g., Klein et al., 2014; Kay et al., 2015; Vo et al., 2017), and changes in decoded neural uncertainty (Liu et al., 2018). Many types of changes at the unit level are consistent with observations at the level of single voxels and across large populations of voxels.“

Regarding simulations, this is an active project in author TCS's lab which will be the topic of a forthcoming publication. The relationship between putative neural mechanisms and observed changes in channel response profiles and stimulus likelihood functions is complex, and a full dissection of this relationship is beyond the scope of the present work, which primarily seeks to illustrate the general points that (1) results from IEM analyses are not arbitrary if the model is considered when interpreting results and (2) some changes in channel response profiles across task conditions may be preserved across arbitrary linear transforms of the IEM basis set. We believe the arguments presented in the manuscript stand with the included simulations. 3. In the last sentence, the authors forgot a citation (Adams, 1978) Good catch!

Reviewer 2: This paper has addressed important theoretical and interpretational issues on the Inverted Encoding Model (IEM) raised by Gardner and Liu (2019), and I believe this paper will contribute to clearing up some confusions about the IEM and stimulate productive debates. We are glad the reviewer thinks our submission will clarify confusions about IEM, and we look forward to future debates inspired by this work. I have some comments about the points that can be strengthened. 1. The authors wrote ”whether an analysis is recovering the hypothetical responses of an arbitrary model rather than assessing the selectivity of population representations is not an issue unique to the inverted encoding model and human neuroscience, but a general problem that must be confronted as more complex analyses intervene between measurement of population activity and presentation of data.“ I strongly agree that all modeling studies are built upon a set of assumptions, which is inevitable given the measurements and the prior knowledge, and the IEM is not unique in that regard. In this context, I think the authors could strengthen their argument if they provided an example(s) from other model-based approaches, such as the Bayesian decoding approach (Van Bergen et al., 2015). For instance, the Bayesian decoding approach also relies on assumptions about the noise covariance structures. Just like the IEM, the authors of the Bayesian decoding

approach used their best prior knowledge about the noise structure, and the results would be inevitably dependent on how the noise structure is assumed in the model. The authors' argument would be more convincing if they showed a concrete example other than the case of an IEM. We thank the reviewer for this suggestion. We agree that all modeling endeavors involve critical assumptions and choices, and those choices can substantially impact the results and conclusions drawn from those results. The noise model choices are an excellent example, and van Bergen et al (2015; 2017) have done an admirable job examining the impact of those choices, especially in the supplement of the 2015 paper. Another concrete example of modeling choices impacting the conclusions drawn from an analysis involves voxel receptive field (vRF) modeling: in early reports using those techniques (e.g., Dumoulin & Wandell, 2008), studies employed a straightforward circular RF model. Subsequent studies expanded on these models, incorporating features like suppressive surrounds (e.g., Zuiderbaan et al, 2012), spatial compressive nonlinearities (e.g., Kay et al, 2013; Mackey et al, 2017), and non-circular RF shapes (e.g., Silson et al, 2018). The conclusions derived from these studies necessarily depend on the features of the model used. Moreover, studies using, e.g., circular RF models in regions where an elliptical model may technically be more accurate are not necessarily 'wrong' - the parameters of those models are still interpretable as the best description of the observed responses given a particular class of model. But, those parameters might change (and variance explained might improve) if a different model is used. We have added discussion of these points to the manuscript. On pg 12:

”Gardner and Liu (2019) also make several other points. First, they highlight many positive aspects of the Bayesian decoding approach introduced by van Bergen et al (2015). We agree - van Bergen et al's (van Bergen et al., 2015; van Bergen and Jehee, 2018) use of a forward model combined with a Bayesian readout rule is an innovative and promising technique, and thoughtfully analyzing data in different ways, especially when employing complex models, is always a good idea. In particular, the Bayesian decoding approach can provide complementary information about the uncertainty with which the activation pattern represents a feature value using an independently-estimated noise model, which is especially useful when trying to link trial-by-trial readouts of neural uncertainty with behavioral measures (van Bergen et al., 2015; Bergen and Jehee, pp2019). That said, we note that the Bayesian approach, like all modeling endeavors, is sensitive to choices made by researchers. For example, analogous to how choices made about channel shapes can impact results from linear IEMs, choices made about the reduced noise model implemented in the Bayesian approach can substantially impact the results (van Bergen and Jehee, 2018). Moreover, invertible linear transforms of the basis set, if not accounted for during the estimation of noise covariance, can lead to large changes in decoding accuracy. As always, motivating analysis choices based on our understanding of neural systems - such as observations that noise covariance scales with tuning similarity (van Bergen and Jehee, 2018) - will enable the most robust possible conclusions.“ On pg 6:

”Moreover, it is often the case that as understanding progresses, decisions about how to specify models change. This is evident among researchers developing and fitting voxel RF models: the original, classical demonstration that RF profiles can be fit for individual fMRI voxels implemented a straightforward circular Gaussian encoding model with an assumption of linear scaling (Dumoulin and Wandell, 2008). In subsequent studies, these models were extended to incorporate inhibitory surrounds (Zuiderbaan et al., 2012), compressive spatial nonlinearities (Kay et al., 2013; Mackey et al., 2017), and noncircular RF profiles (Silson et al., 2018), among many others. Does the introduction of a new model invalidate results using simpler models? Typically not. While it is certainly the case that a more accurate model is always preferable, sometimes a simple model can still be useful (e.g., for defining boundaries of retinotopic ROIs). As always, the parameters reported are interpretable given understanding of the model used to derive those parameters.“

2. The authors listed the strengths and weaknesses of the Bayesian approaches in comparison with the IEM. One of the points they mentioned is that “when trying to disentangle responses associated with simultaneously presented stimuli, specifying an appropriate model in the Bayesian framework is not necessarily straightforward.“ I wish that the authors had clarified why this is particularly problematic for the Bayesian approach and why the IEM would suffer less from the same problem. We apologize for the lack of clarity regarding this point. As described throughout our manuscript (e.g., pg 6 of the resubmission), we consider the IEM a form of model-based directed dimensionality reduction, whereby we transform measurements made in signal (voxel) space into a model-based information (channel) space. Viewed this way, any new measured pattern of activation can be transformed into our modeled channel space (even, in principle, in the absence of a stimulus!). IEM just offers a different (and principled) way to look at neural activation in an experimenter-defined space that can be manipulated and quantified, under a particular set of assumptions. One of those assumptions is that each of several stimulus representations within reconstructed channel response profiles can be assayed independently, which implies some notion of linearity (e.g., Sprague et al, 2014; 2016; 2018). This is not strictly necessary, and other forms of readout/quantification could be constructed that do not require this assumption. Importantly, the linear IEM is most typically fit with a single stimulus 'value' (position, orientation) that does not vary in any properties other than its centroid feature value. This makes the stage of the analysis whereby channel response amplitudes are predicted to generate the design matrix used for computing channel weights relatively straightforward. Specifically, if the model is estimated with a single feature value, you do not need to explicitly write a model for how a channel would aggregate multiple stimuli presented within its range of sensitivity (i.e., its tuning function or receptive field). This is a point of divergence between the linear IEM and the Bayesian framework (van Bergen et al, 2015). Because the Bayesian framework operates on the forward model (it predicts patterns of signals given an estimated encoding model and noise model for any possible stimulus state), it becomes necessary to formalize how multiple stimuli presented together impact neural responses. A simple way to do this (and one used by author TCS in ongoing work; Sprague et al, VSS 2019) is to assume that the response to 2 stimuli is the arithmetic sum of the predicted response to each of the individual stimuli. However, this may be (and likely is!) an incorrect and naïve assumption, and instead a more thorough model incorporating neural encoding properties such as tuned divisive normalization may be necessary. In all likelihood this would involve estimating a more complicated encoding model, perhaps inspired by nonlinear spatial encoding models fit to single-voxel responses in visual and parietal cortex (Kay et al, 2013; Mackey et al, 2017). Moreover, one needs to make a choice about how the noise model might be impacted - do we assume a fixed noise model across conditions? Or do we allow the modeled noise structure to vary across cognitive demands (Cohen & Maunsell, 2009; Mitchell et al, 2009)? These remain open (and fascinating) research questions that we cannot solve here, but we hope the revised commentary makes these issues clear. Finally, we want to emphasize that this need to explicitly describe how multiple stimuli (or stimuli of different sizes, contrasts, etc) are encoded is not necessarily a weakness of the Bayesian method - in fact, it's likely a strength! But it does reduce the simplicity of the analysis, and involves making further assumptions, which will necessarily impact the results (Gardner & Liu, 2019). We've added text to the manuscript (pgs 12-13) to help clarify this important point:

“While the Bayesian approach offers many advantages, there are scenarios where directly comparing responses of modeled information channels is more informative. For example, Brouwer & Heeger (2011) compared responses at specific channels across contrast and stimulus conditions to evaluate the impact of cross-orientation suppression, and Ho et al (2012) and Scolari et al (2012) compared responses in channels tuned nearby the stimulus orientation across task (emphasize speed vs accuracy) and attention (target left vs target right) conditions. These types of analyses require

examining the full response profile across all modeled channels - especially those that are not tuned to the presented stimulus value. This is not easily accomplished with typical decoding analyses - Bayesian or otherwise - that generate a point estimate of the most likely stimulus feature (with or without a corresponding estimate of uncertainty). Moreover, when trying to disentangle responses associated with simultaneously presented stimuli, specifying an appropriate model in the Bayesian framework is not always straightforward. It is explicitly necessary to define a concrete forward model for how multiple stimulus features interact, including, potentially, how they jointly impact the structure of correlated noise. However, this is certainly not a weakness of the Bayesian approach - it is always ideal to explicitly define model assumptions. However, sometimes it can be useful to visualize how activation patterns are altered within a simple and fixed linear model space, in which case the traditional IEM approach can be more appropriate.”

3. The authors mentioned the necessity of using a “fixed encoding model” when comparing different experimental conditions. However, it is not always clear how to build an encoding model that is neutral to all experimental conditions. Even if independent datasets where a different task was performed are acquired for model building, the resulting model could be biased toward one or another condition depending on what cognitive processes are required in the model-building dataset and how similar these processes are to those involved in the main experimental conditions. If we combine the equal amount of data from each experimental condition to build the model, it may still be a problem as the data from the condition with higher SNR are weighted more compared to other condition(s). It would be informative if the authors addressed this issue and suggested a way to mitigate the problem. This is an extremely important point, and one we are actively working on. The reviewer is certainly correct in describing a fundamental issue with the 'fixed encoding model' approach we describe in the commentary: what is the 'neutral' model? When the IEM technique is viewed as a version of model-based directed dimensionality reduction, we believe this concern can be somewhat alleviated. Data from different conditions are projected to different points of a fixed space, and can be directly compared within that space. Of course, how that space is defined is critical. Our overall recommendation is to use the most robust space possible, which typically results from a neutral 'mapping' task with the highest possible SNR. This is similar to the common approach employed when defining retinotopic ROIs using visual stimuli - by presenting the strongest stimulus possible (and sometimes employing attentionally-demanding tasks to further increase signal strength; Bressler & Silver, 2010), experimenters can be confident they're identifying ROIs robustly. However, this might not always be possible, either due to limitations in scan time, or particulars of an experimental manipulation. In this case, it might be a useful alternative to explore the family of model estimation/reconstruction procedures. For example, if each run contains 50% trials from each of two conditions (A and B for simplicity), one could perform leave-one-run-out cross-validation, and estimate 3 models, each used to reconstruct all trials from the held-out run. The first procedure would aggregate trials from both conditions (e.g., Sprague & Serences, 2013), while the other two procedures would involve estimating the model with training trials from only condition A and only condition B, respectively. By comparing the qualitative impact of the manipulation(s) of interest across these model estimation regimes, it is possible to evaluate how data SNR might impact conclusions. In robust scenarios, the impact of the manipulation of interest should be roughly stable across model estimation regimes (while the exact shape of channel response profiles might vary across those regimes, for reasons described eloquently in Liu et al, 2018). We have added a section subtitled “Appropriately selecting neutral training sets” describing the potential impacts of these analysis choices (pg 9):

“It is not always trivial to decide what type of data to use for estimating a fixed encoding model. Data SNR will likely vary across conditions, and fundamental properties of the encoding model itself may also vary (e.g., spatial RF properties, Vo et al., 2017). Because the IEM can be considered a form of model-based dimensionality reduction, it is quite important to estimate the most robust model possible. In general, using a neutral 'mapping' dataset in which data SNR is optimized is, we believe, the most ideal approach, and can provide a stable 'baseline' space in which to transform measured data from an experimental task. For example, when examining how working memory representations change across various task manipulations, we have used a variety of attended visual stimuli for model estimation (Sprague et al., 2016; Rademaker et al., 2019). This is analogous to common procedures used when identifying retinotopic maps on the cortical surface, where researchers typically employ high-contrast flickering checkerboard bars or wedges, and often ask participants to attend the visual stimulus in service of improving SNR (Bressler and Silver, 2010).

It is not always possible to acquire such mapping task datasets, either for experimental or scan time considerations. In this case, there are several possibilities. If multiple conditions are tested (e.g., Conditions 1 and 2), one could (a) estimate an encoding model using data from both Conditions 1 and 2 (equal numbers of trials), then use that encoding model to reconstruct held-out data from each condition (Sprague and Serences, 2013), or (b) estimate the encoding model with data from each condition in turn, then reconstruct data from both Conditions 1 and 2 using each estimated encoding model. The key feature of such an analysis is that channel response profiles are compared based on a fixed encoding model. If different encoding models are used to reconstruct channel response profiles from different conditions, they may not be on equal footing (Liu et al., 2018), and it can be challenging to determine whether the best-fit encoding model changed, the structure of the neural representation changed, or both changed (Sprague et al., 2018a). In general, using a balanced dataset or the highestSNR condition for model estimation should likely yield the most robust results, as these datasets will allow for accurate model estimation.”

4. There are many recent papers on a range of topics using the IEM (see below for a few examples) other than those mentioned in the manuscript. The authors may want to check out these references and include them if relevant. Chen N, Bi T, Zhou T, Li S, Liu Z, Fang F (2015) Sharpened cortical tuning and enhanced corticocortical communication contribute to the long-term neural mechanisms of visual motion perceptual learning. Neuroimage 115:17-29. Chong E, Familiar AM, Shim WM (2016) Reconstructing representations of dynamic visual objects in early visual cortex. Proc Natl Acad Sci USA 113:1453-1458. Lorenc ES, Sreenivasan KK, Nee DE, Vandenbroucke ARE, D'Esposito M (2018) Flexible coding of visual working memory representations during distraction. J Neurosci 38:5267-5276. Thank you for the suggestions. Our original submission was not intended to offer a comprehensive overview of all reports using the IEM technique, and instead we highlight studies most relevant to the points discussed here. Minor point: Page 5: Fig. 2 and Figure 2C should be Fig. 1 and Figure 1C, respectively. Thank you for pointing this out; it's now fixed.

Reviewer 3:

This is an enjoyable response to a valid set of criticisms by Gardner and Liu. Together, the two commentaries (original+response) serve to claify an important set of issues relating to the use of IEMs. I think, frankly, that the two groups are more or less in agreement at this point but this was a useful exchange of views. I wonder if it might be possible for both parties to collaborate on a review paper outlining 'best practises' for the use of IEMs - identifying common pitfalls and illustrating how they can be used in a principled manner for hypothesis testing? We appreciate the reviewer's kind words, and agree that we are essentially in agreement at this point. And that is an excellent suggestion that we will certainly consider.

Reviewer 4: As a response to the commentary by Gardner & Liu (2019), this paper claimed that the inverted encoding model should not be considered arbitrary when the model is properly specified. Even if poorly motivated models are used, differences between conditions are generally preserved. The inverted encoding model remains a useful tool for comparing population-level response profiles across conditions. The clarifications on what can be and what cannot be drawn from the IEM model are important for future research. I hope my questions and comments can help to further enhance its clarity. We are glad the reviewer thinks these clarifications are important for future research. We hope our updated manuscript sufficiently addressed their remaining concerns. 1. The authors clarified that IEM technique is a form of model-based dimensionality reduction, and making inferences about the response properties of single-neurons is not possible using the IEM. How about the inference at a population level? To what extent can the reconstructed response profiles reflect the underlying aggregate neural response change (i.e. baseline change, amplitude change, and bandwidth change) across different conditions? We believe this is exactly the level of analysis for which the IEM framework is best suited. However, it's necessary to qualify: the IEM will be sensitive to population-level changes in response properties at the aggregate level. It remains possible that a complex pattern of response modulations could occur at the single-unit level which aggregate to form a categorically-different form of population-level response modulation. This is well-illustrated for voxel-level receptive fields (vRFs). At the level of single units within a voxel, some neurons might increase their amplitude, others change their baseline, others shrink their RFs, others expand their RFs, and others still shift their preferred position (or, of course, a combination of any/all of the above!). At the aggregate level, these response changes might manifest as a joint change in the position, size, and amplitude of the best-fit vRF for a given voxel. Moreover, at the large-scale population-level (region-level), all these changes across all voxels may support yet another different conclusion about the change in stimulus representations (these issues are discussed in detail in Sprague & Serences, 2013 and Vo et al, 2017). So, results from IEMs should be considered as an assay of the joint representation of a set of aggregate neural responses. But, they cannot necessarily be used to conclude that a given mechanism - even at the aggregate response level - is solely responsible for changes in population-level stimulus representations. If such modulations are the principal research interest, then it would be more ideal to directly compare bestfit encoding models across conditions. While this might be feasible for feature values that are largely represented at a scale commensurate with the measurement modality (e.g., spatial RFs and fMRI voxels), it will be challenging for other features represented at different spatial scales, such as visual orientation. However, labs have employed creative approaches to examine such effects (e.g., Cukur et al, 2013). We address this important issue in the revised Commentary (pg 11):

“Under a fixed encoding model, observing different channel response profiles between task conditions offers meaningful insight into the information content of population-level neural representations. Returning to the point that IEMs act as a form of model-based dimensionality reduction, the observation that channel response profiles change across task conditions suggests that the activation pattern is impacted by the experimental manipulation within the particular modeled information space. For example, manipulations of spatial attention are often found to impact the gain of channel response profiles (e.g., Garcia et al., 2013; Sprague and Serences, 2013; Itthipuripat et al., 2019a). Due to the linearity of the analysis, this is consistent with a neural mechanism whereby neural populations increase their gain with attention (Kim et al., 2007; Lee and Maunsell, 2010; Fig. 1). However, other mechanisms remain possible. As just one example, attention can impact the spatial RFs of neurons or voxels tuned to nearby locations (Womelsdorf et al., 2006), which can also contribute to an observed change in channel response profile amplitude (e.g., Sprague and Serences, 2013; Vo et al., 2017).

Changes in the baseline, width, or even center of channel response profiles can also be meaningfully compared across conditions under a fixed encoding model. For example, Ester et al (pp2019) observed biased centers of channel response profiles following category learning, suggesting that human visual cortex represents similar feature values more distinctly when they belong to different learned abstract categories. Changes in the width of channel response profiles are more challenging to interpret, and less commonly observed, but these could be consistent with a uniform change in the tuning width of constituent neural signals (e.g., simulations in Liu et al., 2018). However, these changes are also consistent with other mechanisms, including asymmetric gain across feature channels (e.g., Scolari et al., 2012).

While it remains impossible to conclusively determine which mechanism(s) at the single neuron level support an observed change in channel response profiles, they can be compared when examined on equal footing using a fixed encoding model. Importantly, similar interpretational issues arise when considering changes in voxel RF parameters across task conditions (e.g., Klein et al., 2014; Kay et al., 2015; Vo et al., 2017), and changes in decoded neural uncertainty (Liu et al., 2018). Many types of changes at the unit level are consistent with observations at the level of single voxels and across large populations of voxels.” And pg 14:

“Finally, Gardner and Liu (2019) and Liu et al (2018) imply that one of the goals of the IEM is to make inferences about single neuron response properties. Making inferences about the response properties of single-neurons is not possible using the IEM or any related model that operates at the scale of aggregate neural signals such as voxels, as different types of single-unit modulations can give rise to identical modulations at the level of a voxel (Sprague et al., 2018a). As described above (”Interpreting channel response profiles“), divergent types of changes in single-neuron response properties can lead to identical signals at the aggregate scale. Thus, making such inferences is not the goal of the IEM or related measures, including the Bayesian decoding approach of van Bergen et al (2015). Instead, a fundamentally different approach that likely requires adopting a different measurement/analysis paradigm, such as parallel characterization of response properties measured across different scales (e.g., fMRI BOLD signal and single-unit electrophysiology; Keliris et al., 2019) would be needed to overcome the ill-posed many-single-neurons-to-voxel mapping problem.”

2. In the section of model comparison, the differences between IEM and Bayesian approaches were not stated clearly. E.g. “These types of analyses require estimating a full response profile across modeled channels, which is not easily accomplished with decoding analyses that generate a point estimate of the most likely stimulus feature....”. What is “full response profile”? Why is it not easily accomplished with decoding analyses? Also, the cases discussed were about the feasibility of IEM

over the Bayesian approach, but not “scenarios when directly comparing responses of modeled information channels can be informative”. We apologize for the lack of clarity in the original submission. We intended to convey that analyses of the type employed by Brouwer & Heeger (2011), Ho et al (2012) and Scolari et al (2012) require comparison of channel response amplitudes for channels that do not correspond to the presented stimulus - that is, you must evaluate the full channel response profile, not just the represented feature value. A Bayesian version of a decoding analysis like that used by van Bergen et al (2015) would not reveal the critical insights afforded by each of these papers. The Bayesian analyses recover a likelihood function describing which stimulus (or stimuli) are likely represented given the observed activation pattern, but this does not provide any information about the response of modeled neural populations tuned to particular feature values relative to the presented stimulus. We disagree with the last sentence of the Reviewer's comment; these are indeed examples where it is necessary to compare the activation of particular modeled information channels, which is also impossible to do in the Bayesian approach. We have clarified this section of the manuscript (pgs 12-13):

“While the Bayesian approach offers many advantages, there are scenarios where directly comparing responses of modeled information channels is more informative. For example, Brouwer & Heeger (2011) compared responses at specific channels across contrast and stimulus conditions to evaluate the impact of cross-orientation suppression, and Ho et al (2012) and Scolari et al (2012) compared responses in channels tuned nearby the stimulus orientation across task (emphasize speed vs accuracy) and attention (target left vs target right) conditions. These types of analyses require examining the full response profile across all modeled channels - especially those that are not tuned to the presented stimulus value. This is not easily accomplished with typical decoding analyses - Bayesian or otherwise - that generate a point estimate of the most likely stimulus feature (with or without a corresponding estimate of uncertainty). Moreover, when trying to disentangle responses associated with simultaneously presented stimuli, specifying an appropriate model in the Bayesian framework is not always straightforward. It is explicitly necessary to define a concrete forward model for how multiple stimulus features interact, including, potentially, how they jointly impact the structure of correlated noise. However, this is certainly not a weakness of the Bayesian approach - it is always ideal to explicitly define model assumptions. However, sometimes it can be useful to visualize how activation patterns are altered within a simple and fixed linear model space, in which case the traditional IEM approach can be more appropriate.”

3. The authors listed a number of modeling approaches, e.g. representational similarity analysis, voxel-wise encoding modeling using naturalistic stimuli, simplified voxel RF modeling using specific stimulus sets. It would be valuable to discuss the applicable scenario for each modeling approach, and also the advantages and disadvantages of IEM over these modeling approaches. We appreciate the suggestion. Overall, we think there are two classes of representational modeling approaches (see also, e.g., Kriegeskorte & Douglas, 2019; Naselaris et al, 2011; Diedrichsen & Kriegeskorte, 2017) (1) those that are interested in assaying the representational geometry of a feature space, for which it is necessary to present stimuli spanning the feature space in order to infer a stable estimate of either the geometry of the feature space and/or the encoding properties relating this geometry to aggregate neural signals, and (2) those that seek to assay properties of population-level stimulus representations, given a previously-estimated encoding model. Both approaches can be employed across conditions, but the second class can compare population-level stimulus representations on single trials. Class 1 includes forward encoding models, like voxel RFs, and representational dissimilarity analyses. Class 2 includes the linear inverted encoding model as well as the Bayesian decoding method of van

Bergen et al (2015). One could also lump more traditional multivariate decoding analyses (e.g., multi-class categorical decoding) within this class as well. We think the biggest point of distinction between these types of models is that Class 1 necessitates estimation of a full encoding model (or representational dissimilarity matrix) for each task condition of interest, which necessarily requires presenting a wide array of visual stimuli across trials or extended measurement intervals, while Class 2 compromises the accuracy of the best-fit encoding model in exchange for the ability to directly compare population-level stimulus representation across task conditions or even across single trials. We have expanded our discussion of these other methods in a new section subtitled “Instantaneous decoding vs extended model estimation” in the revised Commentary (pgs 13-14):

“Here, we have primarily discussed the IEM technique and a Bayesian decoder based on a formally specified encoding model. There are several other techniques that have been applied to understand neural representations, including representational similarity analysis (RSA; Kriegeskorte et al., 2008; Kriegeskorte and Kievit, 2013), voxel-wise encoding modeling (Kay et al., 2008; Naselaris et al., 2009, 2011; Nishimoto et al., 2011; Huth et al., 2012, 2016; Çukur et al., 2013; Lescroart and Gallant, 2019) and voxel receptive field (RF) modeling (Dumoulin and Wandell, 2008; Kay et al., 2013; Wandell and Winawer, 2015; Vo et al., 2017). These approaches can be broken down based on their goals: do we wish to build a comprehensive model of a stimulus' representational space within a brain region, including the sensitivity profiles of constituent voxels? Or, do we instead prefer using a simplified model to make inferences about 'instantaneous' brain states that can be compared across experimental conditions?

RSA, simplified vRF modeling, and voxel-wise modeling all require participants view stimuli spanning an entire stimulus space to estimate a single result (for RSA: a representational dissimilarity matrix; for voxel-wise modeling, the parameters to each voxel's encoding model). For example, you cannot estimate a voxel's RF profile with stimuli presented in a small number of positions (e.g, just one position on the left and one on the right side of the screen). Instead, you must present stimuli that span the entire stimulus space (e.g., the full extent of the screen, from top to bottom and side to side). Thus, to compare results from any of these methods between task conditions, one must exhaustively sample the stimulus space within each task condition independently. Several studies have taken this approach. Çukur et al. (2013) fit voxel-wise encoding models of semantic space to data acquired while participants viewed natural movies and reported the presence of faces or vehicles (in different scan runs). Accordingly, between scan runs, they could compare properties of the best-fit encoding models across voxels, and concluded that the structure of semantic space is warped between conditions. Several studies have manipulated the locus of spatial attention while participants view visual stimuli used to map vRFs. Klein et al (2014) and Vo et al (2017) required participants attend to fixed locations while presenting mapping stimuli, and Sprague & Serences (2013), Kay et al (2015), Sheremata & Silver (2015), and van Es et al (2018) all estimated voxel RF profiles during scanning runs when participants either attended to or ignored the mapping stimuli.

RSA and voxel-wise encoding models stand in contrast to the IEM, the Bayesian decoding method, and other decoding approaches. Using these methods, once an encoding model or decoder is estimated (on a held-out set of data), it can be applied to any new activation pattern, even on a timepoint-by-timepoint basis. One case in which this can be used is to compare channel response profiles across a large number of conditions, such as the location of spatial attention and stimulus contrast (Itthipuripat et al., 2019a). In this study, it was not necessary to evaluate channel response profiles at many different stimulus positions, so instantaneous estimates for a small number of stimulus positions offered an efficient means to determine the joint impacts of attention and contrast on population-level stimulus representations. Put another way: there is no way to estimate a voxel-wise encoding model, vRF profile, or representational dissimilarity matrix on a single trial. If single-trial

analyses are critical for a given research question, applying an independently-estimated encoding model or decoder is necessary. If, instead, a careful assay of the representational geometry and/or encoding properties of a neural signal are important, analysis of best-fit encoding models to extended datasets should be used.

Finally, the two approaches might be integrated: if trying to understand how a poorly-understood feature space is represented on individual trials, it may be best to start by inferring representational geometry with modern versions of techniques like RSA (Kriegeskorte et al., 2008; Kriegeskorte and Kievit, 2013; Walther et al., 2016; Cai et al., 2019), then using features of the inferred geometry in combination with model-based analysis methods like IEM or a Bayesian decoder applied to a separate dataset to recover trial-by-trial representations of feature information.”
